# The associations between cerebral microhemorrhages and cognitive decline across Alzheimer’s continuum

**DOI:** 10.1007/s40520-025-02988-8

**Published:** 2025-05-13

**Authors:** Homayoon Khaledian, Ali Julaee Rad, Pardis Barjisi, Parsa Saberian, Mehrdad Mozafar, Sahar Ghahramani, Mohammad Sadeghi, Mahsa Mayeli, Seyed Mohammad Amin Alavi, Soorin Berenjian, Shaghayegh Karami, Mohadeseh Andalibian

**Affiliations:** 1 Borderless Research, Advancement and Innovation in Neuroscience Network (BRAINet), Tehran, Iran; 2 Department of Neurosurgery, Faculty of Medicine, Tehran Medical Sciences Branch, Islamic Azad University, Tehran, Iran; 3 Master’s Student, Department of Psychology, Tehran University, Tehran, Iran; 4 Department of Biomedical Engineering, Islamic Azad University of Urmia, Urmia, Iran; 5 Faculty of Medicine, Hormozgan University of Medical Sciences, Bandar Abbas, Iran; 6 School of Medicine, Tehran University of Medical Sciences, Tehran, Iran; 7 Health Policy Research Center, Shiraz University of Medical Sciences, Shiraz, Iran; 8 School of Rehabilitation, Shiraz University of Medical Sciences, Shiraz, Iran; 9 Faculty of Medicine, Ahvaz Jundishapur University of Medical Sciences, Ahvaz, Iran; 10 Department of Psychology, Islamic Azad University of Isfahan (Khorasgan) Branch, Isfahan, Iran; 11 Department of psychology, Hakim Toos Institute of Higher Education, Mashhad, Iran; 12 Hormozgan University of Medical Sciences, Chamran St, Bandar Abbas, 79169-69573 Iran

**Keywords:** Cerebral microhemorrhages, Cognitive decline, Alzheimer's disease, Mild cognitive impairment, T2-weighted MRI

## Abstract

**Objective:**

To investigate the associations between cerebral microhemorrhages (CMH) and cognitive decline across the Alzheimer’s dementia continuum.

**Methods:**

Using the Alzheimer’s Disease Neuroimaging Initiative (ADNI) database, we studied 619 participants, categorized into 221 cognitively normal (CN) participants, 281 patients with mild cognitive impairment (MCI), and 117 patients with Alzheimer’s disease (AD). CMH prevalence and distribution were determined using T2-weighted magnetic resonance imaging (MRI), focusing on the frontal, occipital, and parietal subcortical regions of interest (ROIs).Clinical dementia rating scale sum of boxes (CDR-SB) and mini-mental state examination (MMSE) were used for diagnosis and composite cognitive scores regarding visuospatial abilities, language, memory, and executive functions were used as outcome variables. Age, gender, and APOE ε4 positivity status were used as covariates.

**Results:**

The AD group displayed significantly elevated tau and P-tau levels compared to MCI and CN groups (*p* < 0.001). APOE ε4 positivity was 67.5% in the AD group, surpassing the 50.2% in MCI and 29% in CN individuals (*p* < 0.001). Cognitive assessments revealed that the AD group’s CDR-SB score and MMSE both significantly differed from these scores in the MCI and CN groups (*p* < 0.001). Overall, CMH prevalence was 27.7%, with a predominant distribution in the frontal subcortical ROIs. MCI subjects with CMH showed notably diminished ADNI Visuospatial Composite Scores compared to those without CMH. Age significantly predicted CMH in CN and MCI (*p* < 0.05). In AD participants, APOE ε4 heterozygotes (*p* = 0.02) and homozygotes (*p* = 0.01) hadincreased CMH likelihood.

**Conclusion:**

CMHs are significantly associated with cognitive decline in patients with MCI. This association is more prominent in regard to the decline in visuospatial abilities.

## Introduction

Dementia is a medical condition characterized by the deterioration of cognitivefunctions. It is mostly caused by several neurodegenerative conditions, with Alzheimer’s disease (AD) being the most prevalent [1]. AD is a neurological condition that often begins with memory problems and cognitive decline. Over time, it can also impact behavior, speech, visuospatial orientation, and the motor system [2]. With the aging of the global population, it is projected that the frequency will double by 2030 and triple by 2050 [3].

Vascular pathologic processes are crucial in cognitive decline [4]. The lesions can be categorized as either ischemic, which includes lacunes and white matter lesions, or hemorrhagic. Cerebral microbleeds that are distributed in a lobar pattern are considered to be indicators of cerebral amyloid angiopathy [5]. They are frequently observed in individuals with AD [6–8].

Various forms of imaging have been used to evaluate cerebral microhemorrhages (CMH). Magnetic resonance imaging (MRI) can identify MCH and predict the likelihood of spontaneous rebleeding or bleeding problems after anticoagulation [9]. Until recently, different imaging approaches in terms of number and mode of propagation were used to study MCH. Kato et al. employed gradient-echo T2*-weighted MRI to show the prevalence and number of MCH [10].

Due to the association between the lobar distribution of MCH and AD, detecting MCH is critical for identifying numerous disorders, including dementia and AD [11, 12]. Based on previous literature, those who have numerous MCH are more likely to acquire a more severe form of dementia over time [13]. Although, previous studies suggest that MCHs affect cognitive function, and their presence is linked to worse cognitive performance, the connection between MCH presence, progression, quantity, and location with cognitive decline manifestations is still not well understood [14, 15].

Using the Alzheimer’s Disease Neuroimaging Initiative (ADNI) data, we aimed to examine the associations between microhemorrhage findings on weighted images and cognitive decline across the AD spectrum.

## Methods

### Study design and participants

Data used in this research was extracted from the Alzheimer’s Disease Neuroimaging Initiative (ADNI).

1216 participants with baseline demographics, CSF biomarkers, and cognitive scores were screened. The inclusion criteria were clinical diagnosis of normal cognition (CN), MCI, or AD, age over 60, and available imaging and cognitive evaluation data. We excluded patients with cavernous, venous, and macro hemorrhagic infarction patients and included only “definite” CMH patients. Eventually, a total of 619 patients were included in the analysis. All data is available upon request on ADNI (http://adni.loni.usc.edu).

### Measurement of biomarkers

Biomarker measurements were conducted using standardized procedures. The CSF biomarkers analyzed in this study included Aβ1–42, t-tau, and p-tau181. The measurements were performed using the highly validated Roche Elecsys cobas e 601 fully automated immunoassay platform and reference LC/MSMS methodology for CSF Aβ1–42, Aβ1–40, and Aβ1–38 [16, 17]. A complete information regarding the imaging protocol can be found on the ADNI website (http://adni.loni.usc.edu).

### Imaging protocol

T2-weighted gradient echo images sensitized for local iron deposits were used. MRI parameters were optimised to acquire high quality images. In a 3 mm plane, image resolution was 1 mm. Pictures were registered and resampled to identify microhemorrhages. A built in probability atlas was used. The T1-weighted images were registered to the T2 images and resampled into display space using cubic-spline interpolation. An expert analyst or radiologist identified anomalies in the observation image as possible, definite, or withdrawn. Image intensity rarely needed manual adjustment [18]. ADNI’s imaging methodology is available at http://adni.loni.usc.edu.

### Cognitive assessments

Several cognitive assessments were utilized, including the Clinical Dementia Rating Scale-Sum of Boxes (CDR-SB) and Mini-Mental State Exam (MMSE). Furthermore, the data of four ADNI composite scores, including ADNI-memory (ADNI-MEM), ADNI-executive function (ADNI-EF), ADNI-language (ANDI-LAN), and ADNI-visuospatial (ADNI-VS) were used in this investigation. Complete information on the aforementioned composite scores was previously published [19–21]. Raw composite scores can be found on the ADNI website (http://adni.loni.usc.edu).

### Statistical analyses

Chi-square tests were used to evaluate categorical variables, while Kolmogorov-Smirnov tested continuous variables for normal distribution. One-way ANOVA and Kruskal-Wallis was used for normally and non-normally distributed continuous variables. CMH predictors in CN, MCI, and AD groups were investigated using logistic regression. Subgroup analyses are done for each CMH group. CMH-related cognitive assessments were compared across brain areas using one-way ANOVA. All statistical analyses was conducted using IBM SPSS ver. 26 (IBM Corp, Armonk, NY, USA) with a significance level of *p* = 0.05.

## Results

### Demographic characteristics

A total of 619 individuals were included in the final analysis. The study population consisted of 221 cognitively normal (CN) individuals, 281 patients with MCI, and 117 patients with AD. The mean age was 72.58 ± 7.15 years, and the mean education level was 16.35 ± 2.59 years. The female-to-male ratio was 288 to 331. Demographic and baseline characteristics for each group are presented in Table 1.

**Table 1:** Demographic and background characteristics of study population

Variable	CN (N = 221)	MCI (N = 281)	AD (N = 117)	p-value	Post-hoc (Bonferroni)
Age	72.84 ± 6.09	71.73 ± 7.24	74.10 ± 8.42	< 0.01	AD > MCI
Gender(female/male)	53.4%/46.6%	43.1%/56.9%	41.9%/58.1%	0.03	CN
Education	16.57 ± 2.51	16.41 ± 2.62	15.77 ± 2.64	0.02	CN > AD
APOE E4 (positive/negative)	29%/71%	50.2%/49.8%	67.5%/32.5%	< 0.001	CN, MCI, AD
Tau	237.46 ± 91.33	281.54 ± 132.46	377.51 ± 155.16	< 0.001	AD > MCI > CN
Ptau	21.70 ± 9.48	27.25 ± 14.83	37.23 ± 16.11	< 0.001	AD > MCI > CN
CDRSB	0.04 ± 0.15	1.51 ± 0.88	4.59 ± 1.71	< 0.001	AD > MCI > CN
MMSE	29.02 ± 1.24	28.01 ± 1.76	23.13 ± 2.09	< 0.001	CN > MCI > AD
ADNI-MEM	1.09 ± 0.59	0.24 ± 0.70	-0.89 ± 0.53	< 0.001	CN > MCI > AD
ADNI-EF	0.89 ± 0.84	0.32 ± 0.91	-0.84 ± 0.98	< 0.001	CN > MCI > AD
ADNI-LAN	0.85 ± 0.70	0.29 ± 0.78	-0.72 ± 0.92	< 0.001	CN > MCI > AD
ADNI-VSP	0.22 ± 0.58	-0.09 ± 0.75	-0.62 ± 0.95	< 0.001	CN > MCI > AD
MCH (yes/no)	20.8%/79.2%	31%/69%	33.3%/66.7%	0.01	CN

Abbreviation: CDRSB; clinical dementia rating scale sum of boxes, MMSE; mini-mental state exam, ADNI-MEM; ADNI memory composite score, ADNI-EF; ADNI executive function composite score, ADNI-LAN; ADNI language composite score, ADNI-VSP; ADNI visuospatial composite score, MCH; micro cerebral hemorrhage

Continuous and categorical variables are presented as mean ± standard deviation and percentage, respectively

### AD biomarkers

Biomarker measurements revealed significant differences among the groups. The AD group exhibited higher levels of tau (377.51 ± 155.16 pg/mL) and phosphorylated tau (p-tau) (37.23 ± 16.11 pg/mL) compared to the MCI group and the CN group (*p* < 0.001). APOE ε4 positivity was found to be significantly associated with disease status, with higher percentages of APOE ε4 positivity observed in the AD group (67.5%) compared to the MCI group (50.2%) and the CN group (29%) (*p* < 0.001). Complete details of all scores can be found in Table 1.

### Cognitive assessments

The AD group had higher CDR-SB scores (4.59 ± 1.71) and lower MMSE scores (23.13 ± 2.09) compared to the MCI and CN groups (p-value < 0.001). Notably, the CN group showed the highest scores in in cognitive domains, including ADNI-MEM, ADNI-EF, ADNI-LAN, and ADNI-VSP, followed by the MCI group, and the AD (*p* < 0.001 for all comparisons). Complete details of all scores can be found in Table 1.

### Imaging findings

**Fig. 1:**
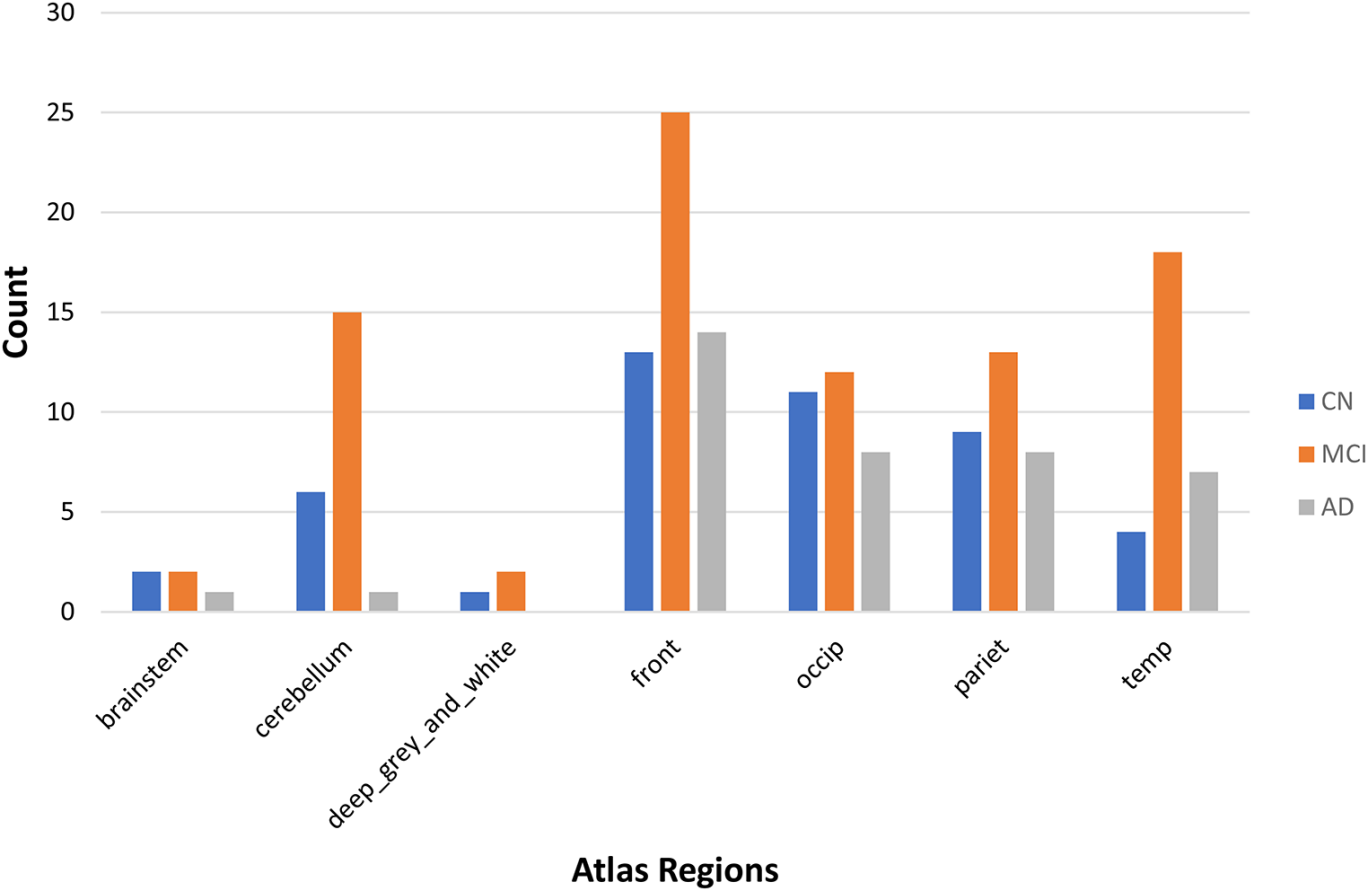
Distribution of micro cerebral hemorrhage (MCH) sites among each group

The prevalence of CMH in the overall population was 27.7% (172 out of 619 participants), including 46 CN individuals (20.8%), 87 MCI patients (31%), and 39 AD patients (33.3%). The distribution of microhemorrhages detected with T2-weighted MRI showed that the frontal subcortical region exhibited the most occurrences (25 microhemorrhages in the MCI group and 14 microhemorrhages in the AD group), followed by the occipital subcortical region (12 microhemorrhages in MCI, 11 in CN, and 8 in AD), and the parietal subcortical region (13 microhemorrhages in MCI, 9 in CN, and 8 in AD). Detailed information on CMH distribution across groups is illustrated in Fig. 1.

Regarding the association between CMH and cognitive decline, MCI patients with CMH exhibited significantly lower scores in the ADNI-VS Composite Score (mean: -0.25 ± 0.76) compared to MCI patients without CMH (mean: -0.02 ± 0.74), indicating a more pronounced impairment in visuospatial abilities (Table 2).

**Table 2:** Cognitive assessments comparison between subjects with/without MCH for each group

Variables	CN			MCI			AD		
	Without MCH	With MCH	P value	Without MCH	With MCH	P value	Without MCH	With MCH	P value
**CDRSSB**	0.04 ± 0.14	0.04 ± 0.17	0.6	1.42 ± 0.81	1.7 ± 1.01	0.05	4.57 ± 1.65	4.62 ± 1.85	0.99
**MMSE**	29.01 ± 1.23	29.09 ± 1.26	0.6	28.14 ± 1.69	27.72 ± 1.9	0.08	23.21 ± 2.21	22.97 ± 1.84	0.5
**ADNI-MEM**	1.10 ± 057	1.06 ± 0.68	0.66	0.28 ± 0.73	0.16 ± 0.62	0.17	-0.86 ± 0.56	-0.95 ± 0.47	0.39
**ADNI-EF**	0.87 ± 0.82	0.94 ± 0.92	0.64	0.38 ± 0.89	0.2 ± 0.94	0.12	-0.84 ± 1.03	-0.83 ± 0.86	0.95
**ADNI-LAN**	0.83 ± 0.68	0.92 ± 0.77	0.42	0.34 ± 0.77	0.2 ± 0.81	0.19	-0.64 ± 0.95	-0.88 ± 0.84	0.18
**ADNI-VSP**	0.2 ± 0.59	0.26 ± 0.55	0.62	-0.02 ± 0.74	-0.25 ± 0.76	**0.01**	-0.63 ± 0.96	-0.58 ± 0.95	0.79

Abbreviation: CDRSB; clinical dementia rating scale sum of boxes, MMSE; mini-mental state exam, ADNI-MEM; ADNI memory composite score, ADNI-EF; ADNI executive function composite score, ADNI-LAN; ADNI language composite score, ADNI-VSP; ADNI visuospatial composite score, MCH; micro cerebral hemorrhage

Variables are presented as mean ± standard deviation

Significant values are presented in bold formats

*P* values for CDRSB, MMSE and ADNI-VS variables were resulted from *Mann-Whitney* analyses, otherwise *Two independent T test* was conducted

Logistic regression analyses showed that age was a significant predictor of CMH in both the CN and MCI groups, as aging was associated with increased odds of CMH (OR = 0.89 and 0.92 for CN and MCI, respectively; *p* < 0.05). In the AD group, the presence of APOE ε4 heterozygotes (OR = 0.24, p-value = 0.02) and homozygotes (OR = 0.14, p-value = 0.01) were associated with increased chances of CMH. Table 3 represents detailed information on predicting the effect of other variables.

**Table 3:** **Result from Logistic regression analyses conducted for possible predictors of MCH among each group.**

Variables	CN			MCI			AD		
	Beta	**Odds ratio**	P value	Beta	Odds ratio	P value	Beta	Odds ratio	P value
**Age**	-0.11	0.89	**0.01**	-0.07	0.92	**0.002**	-0.05	0.94	0.08
**Gender**	-0.55	0.57	0.22	0.18	1.2	0.56	-0.82	0.44	0.12
**APOE4**									
Heterozygote	0.51	1.67	0.31	-0.24	0.77	0.48	-1.42	0.24	**0.02**
Homozygote	1.17	3.24	0.4	-0.42	0.65	0.42	-1.96	0.14	**0.01**
**ABETA**	0.00	1	0.13	0.00	1	0.74	-0.00	0.99	0.68
**TAU**	0.01	1.01	0.31	0.00	1	0.95	0.01	1.01	0.23
**pTau**	-0.12	0.88	0.34	-0.007	0.99	0.89	-0.15	0.85	0.08
**CDRSB**	-0.58	0.55	0.68	-0.29	0.74	0.08	-0.04	0.95	0.77
**MMSE**	0.16	1.17	0.42	0.12	1.13	0.19	-0.04	0.95	0.76
**ADNI-MEM**	-0.6	0.54	0.18	-0.45	0.63	0.15	0.63	1.88	0.51
**ADNI-EF**	-0.4	0.66	0.26	-0.07	0.92	0.75	-0.23	0.37	0.53
**ADNI-LAN**	0.01	1.01	1.01	-0.1	0.9	0.69	0.32	1.38	0.4
**ADNI-VS**	0.17	1.19	0.64	0.25	1.29	0.21	0.1	1.1	0.73

Abbreviation: CDRSB; clinical dementia rating scale sum of boxes, MMSE; mini-mental state exam, ADNI-MEM; ADNI memory composite score, ADNI-EF; ADNI executive function composite score, ADNI-LAN; ADNI language composite score, ADNI-VSP; ADNI visuospatial composite score, MCH; micro cerebral hemorrhage

Significant values are presented in bold formats

Note: Presence and absence of MCH have been coded as 0 and 1 in ADNI dataset, respectively. Therefore, negative B values indicating that by increasing the value there is greater chance of MCH

Subgroup analyses were conducted on participants with CMH, including 172 individuals (46 CN, 39 MCI, and 87 AD). In each group, no significant differences were observed in any of the cognitive variables between brain regions with MCH.

## Discussion

The current investigation aimed to examine the correlations between the presence of microhemorrhages and the development of cognitive decline across the AD continuum. Tau biomarkers have gained traction as potential indicators for AD. previous studies highlighted significant differences in CSF tau biomarkers across CN, MCI, and AD patients [22, 23]. In the current investigation, we observed discernible differences among the groups, with the AD group showing elevated levels of tau and p-tau in comparison to both the MCI and CN groups (*p* < 0.001). Taken together, these findings reaffirm the diagnostic potential of tau biomarkers in distinguishing AD from other cognitive conditions. Prior research has illustrated the predictive capability of APOE ε4 status in relation to various stages of disease [24, 25]. In alignment with previous findings, our study revealed a substantial association between APOE ε4 positivity and disease status. Notably, we observed higher proportions of APOE ε4 positivity within the AD group (67.5%) in contrast to both the MCI group (50.2%) and the CN group (29%), with the differences being statistically significant (*p* < 0.001). The mentioned evidence collectively underscores the potential utility of APOE4 as an informative marker for understanding and categorizing different stages of cognitive impairment and AD.

Lynch et al. (2006) observed that individuals with MCI, despite having the same global CDR score of 0.5 as those with very mild AD, presented with a notably lower CDR-SB score [26]. O’Bryant et al. (2008) studied 1,577 participants and found a significant difference in CDR-SB scores among CN, MCI, and AD patients. Their findings not only demonstrated the diagnostic potential of CDR-SB in staging AD severity but also emphasized its utility in clinical settings [27]. Moreover, MMSE, another widely used diagnostic tool, showcased consistent findings. As elucidated by Kato et al. (2013), AD patients exhibited poorer performance on the MMSE relative to both CN and MCI groups, even after adjusting for potential confounders like age and gender [28]. Collectively, these findings underscore the significance of these assessments in distinguishing between CN, MCI, and AD groups, potentially guiding clinicians in diagnosing and staging the severity of cognitive decline in their patients. In our study, we observed that individuals in the AD group displayed higher scores on the CDR-SB assessment, with an average score of 4.59 ± 1.71, which was similar to previous investigations. Conversely, both MCI and CN groups exhibited lower CDRSB scores. The difference in scores between the AD group and both the MCI and CN groups was statistically significant (p-value < 0.001). Additionally, the AD group demonstrated lower scores on the MMSE, with an average score of 23.13 ± 2.09, further indicating a higher cognitive impairment compared to the MCI and CN groups.

Our investigation evaluated the prevalence of CMHs in AD, MCI, and CN groups and determined that no significant differences between these groups. The distribution of regions with microhemorrhages detected with T2-weighted MRI were also investigated, and findings revealed that the MCI group exhibited the highest occurrence in subcortical areas. However, a study by Yates et al. (2014) revealed that Lobar microbleeds (LMBs) were observed in a higher percentage of MCI and AD participants compared to NC participants (*p* < 0.05 vs. NC) [29]., which was opposed to the current study’s findings. We identified (LMBs) or MCH, particularly those located in the temporal and frontal lobes, as having a more pronounced impact on cognitive decline. MCHs were also much more prevalent in lobar areas among the MCI group (Fig. 1). The study by Li et al. (2020) investigated the association between MCH and cognitive decline over time [14]. They found that the presence, progression, and higher number of MCH were associated with cognitive deterioration, particularly in memory, executive function, and global cognitive function. The study highlighted that lobar MCH, especially those located in the temporal lobe, played a major role in the relationship between MCH and cognitive decline. The results of current the investigation revealed an association between MCH and cognitive decline among MCI individuals. MCI individuals with MCH scored significantly lower in the ADNI-VS Composite Score compared to MCI patients without MCH. Previous literature illustrated that individuals suffering from incident dementia and MCH exhibited a more significant decline in visuospatial skills compared to those without MCH [30], which was similar to the current study’s findings.

In our study the Logistic regression analyses showed that age was a significant predictor of MCH in both the CN and MCI groups, as aging was associated with increased odds of MCH (OR = 0.89 and 0.92 for CN and MCI, respectively; *p* < 0.05). In cross-sectional studies, LMBs have been associated with increasing age. Poels et al. (2010) showed that the prevalence of microbleeds demonstrated a gradual rise as individuals grew older, starting from 6.5% in people aged 45 to 50 years and reaching 35.7% in participants aged 80 years and above [31]. Moreover, Yates et al. (2014) stated that older age may increase the risk of incident lobar microbleeds [29].

Our research revealed an association between the presence of APOE ε4 in both heterozygotes and homozygotes, and increased likelihood of CMH. In contrast, Stefaniak et al. (2018) and Sepehry et al. (2016) found that the carrier status of APOE ε4 was not notably linked to the definitive presence of CMH [32, 33].

### Limitations

While our study offers valuable insights into the association between CMH and cognitive decline across the AD continuum, several limitations should be acknowledged. Firstly, the cross-sectional design provides only a snapshot of the data, limiting our ability to infer causality or the progression of cognitive decline over time. Future longitudinal studies are required to assess the temporal relationship between CMH and cognitive decline, determining whether CMH predicts cognitive deterioration over time. Additionally, while our study controlled for age and APOE ε4 status, other potential confounding factors such as hypertension, diabetes, and small vessel disease were not accounted for, which may influence CMH occurrence and cognitive impairment. Finally, the unequal sample sizes across groups, particularly the smaller AD cohort, may limit the robustness of subgroup analyses.

## Conclusion

Our study identifies an association between CMH and cognitive decline, particularly in visuospatial abilities among individuals with MCI. MCI patients with CMH exhibited significantly lower ADNI-VS Composite Scores compared to those without CMH, suggesting a potential link between CMH and more pronounced visuospatial impairment. Additionally, MCH prevalence was higher in the AD and MCI groups, with age emerging as a significant predictor of CMH in cognitively normal and MCI individuals. Among AD participants, the presence of APOE ε4 alleles was associated with an increased likelihood of CMH. While our findings contribute to understanding CMH across the Alzheimer’s dementia continuum, future longitudinal studies are necessary to establish the temporal and causal relationships between CMH and cognitive decline. Further research is also needed to explore the underlying mechanisms and potential clinical implications of CMH in AD.

## Data Availability

Data used in this research is available upon request at https://adni.loni.usc.edu/.

## Declarations

### Presentation

N/A

### Generative AI in scientific writing

The writers express their recognition of utilizing a Large Language Model (LLM) during the creation of this publication. ChatGPT, a language model created by OpenAI (version: GPT-4), was used to help in the process of writing, revising, and improving the text. The authors have thoroughly examined and verified the content to guarantee its precision and authenticity. The writers assume complete accountability for the factual precision, citation consistency, mathematical accuracy, logical consistency, and overall novelty of the article.

## Abbreviations

ADNI: Alzheimer’s Disease Neuroimaging Initiative
CMH: Micro cerebral hemorrhage
AD: Alzheimer’s disease
MCI: Mild cognitive impairment
CN: Cognitively normal
MMSE: Mini-Mental State Examination
